# Epsins Regulate Mouse Embryonic Stem Cell Exit from Pluripotency and Neural Commitment by Controlling Notch Activation

**DOI:** 10.1155/2019/4084351

**Published:** 2019-02-25

**Authors:** Marina Cardano, Jacopo Zasso, Luca Ruggiero, Giuseppina Di Giacomo, Matteo Marcatili, Ottavio Cremona, Luciano Conti

**Affiliations:** ^1^Laboratory of Stem Cell Biology, Department of Cellular, Computational and Integrative Biology (CIBIO), Università degli Studi di Trento, Trento, Italy; ^2^Università Vita Salute San Raffaele, Ospedale San Raffaele, Division of Neuroscience, Milan, Italy; ^3^Pediatric Highly Intensive Care Unit, Fondazione IRCCS Ca' Granda Ospedale Maggiore Policlinico and University of Milan, Milano, Italy

## Abstract

Epsins are part of the internalization machinery pivotal to control clathrin-mediated endocytosis. Here, we report that epsin family members are expressed in mouse embryonic stem cells (mESCs) and that epsin1/2 knockdown alters both mESC exits from pluripotency and their differentiation. Furthermore, we show that epsin1/2 knockdown compromises the correct polarization and division of mESC-derived neural progenitors and their conversion into expandable radial glia-like neural stem cells. Finally, we provide evidence that Notch signaling is impaired following epsin1/2 knockdown and that experimental restoration of Notch signaling rescues the epsin-mediated phenotypes. We conclude that epsins contribute to control mESC exit from pluripotency and allow their neural differentiation by appropriate modulation of Notch signaling.

## 1. Introduction

Endocytosis is a conserved process by which plasma membrane components, extracellular molecules, and macromolecular complexes are internalized in the cell after being segregated into vesicles by a highly specialized machinery. Endocytosis is crucial to regulate the dynamics and composition of plasmalemmal components, thus critically controlling the communication with the extracellular environment. Although there are various mechanisms of endocytosis, internalized vesicles are delivered to a common specialized membrane compartment, the endosome, from where endocytosed molecules are sorted into different vesicle trafficking pathways for recycling, degradation, or rerouting [1].

Given its essential role in plasma membrane homeostasis and in extracellular signaling, endocytosis has to be continuously adapted to the cell's functional state. Among the various players which critically regulate the endocytic process, epsins (EPNs) function as scaffolds which bring together structural and accessory factors of the endocytic machinery, phosphoinositides, and ubiquitylated cargos for internalization [2–4]. EPN orthologues in yeast have been shown to function not only in endocytosis but also in cell polarity through an endocytosis-independent regulation of actin dynamics [5]. In invertebrates, EPNs have shown a critical role for ubiquitin-dependent synaptic growth [6] and Notch pathway activation [7–11]. The versatility in EPN functions can be justified by their unique multimodular structure, consisting of (i) an N-terminal ENTH domain for plasma membrane anchoring to phosphoinositides [12, 13], (ii) multiple ubiquitin-interacting motifs (UIMs) for ubiquitin recognition and EPN ubiquitination [14], (iii) a clathrin/AP2-binding domain [15, 16], and (iv) multiple C-terminal NPF motifs for the recognition of EH domain containing proteins [12]. Currently, the EPN family includes three members: epsin1 and epsin2 (EPN1 and EPN2), which are ubiquitously expressed [3, 12, 17], and epsin3 (EPN3), which seems to be restricted to surface epithelia [18]. The simultaneous ablation of the three EPNs generates *in vitro* severe cell division defects associated with impairment of clathrin-mediated endocytosis [19]. Moreover, genetic studies in mice have demonstrated that ablation of both EPN1 and EPN2 results in arrest of embryo development at midgestation, with multiorgan defects tightly recapitulating those observed in mutants of the Notch signaling pathway [17]. Particularly, the EPNs' role in Notch signaling activation critically depends on ubiquitin-mediated internalization of Notch ligands. Indeed, the mechanical traction exerted by EPN-triggered endocytosis on the Notch ligands (in the sending cell) during endocytosis is required to activate the proteolytic cleavage of the Notch receptors (in the receiving cell), which ultimately releases the Notch intracellular domain (NICD), the effector of Notch signaling in the nucleus [7–9, 20–22]. Notably, EPN ENTH domain association with the plasma membrane, by lowering plasmalemma bending rigidity, should make the pulling action exerted by EPNs in endocytosis energetically even less costly [23].

Although EPNs have been extensively studied in organogenesis, multipotent and adult stem cells, their possible function in pluripotent stem cells is rather unexplored. Here, we investigated the role of EPNs on mouse embryonic stem cells (mESCs) biology and we found that knockdown (KD) of both EPN1 and EPN2 stimulates mESC pluripotency exit and differentiation. Additionally, we found that these defects are associated with a specific impairment of Notch activation. Downstream targets of Notch signaling were also misregulated by EPN silencing, producing Notch KO-like phenotypes during neural differentiation, which were rescued by NICD overexpression.

Collectively, these results suggest that EPN-mediated endocytosis plays a critical role in controlling appropriate activation of Notch signaling in mESCs allowing their proper exit from pluripotency and differentiation.

## 2. Material and Methods

### 2.1. Cell Culture and Transfection

All the mESC lines used in this study (46C [24], E14, *β*III-tubulin::eGFP [25], and OG2 [26]) were cultured in feeder-free conditions on a 0.1% gelatin-coated plastic in GMEM supplemented with 10% FBS, 2 mM L-glutamine, 1 mM sodium pyruvate, nonessential amino acids, 100 *μ*M 2-mercaptoethanol (Thermo Fisher Scientific), and 1000 units/ml LIF (Millipore). Cells were passaged by trypsinization every 2-3 days at a 1 : 6-1 : 8 ratio. ESC colony-forming assay was carried out by plating 100 mESCs per cm^2^ on 0.1% gelatin-coated plates. After 5 days, cells were fixed and stained for alkaline phosphatase (AP) (Millipore, cat. SCR004), following the manufacturer's protocol, and images were collected with a Leica DM IL Led microscope provided of a Leica DFC450 C camera (Leica Microsystem). The number of the positive colonies and their size were quantified by ImageJ software. Embryoid body assay was performed by the hanging drop methods. Briefly, mESCs were resuspended in GMEM medium and 100 *μ*M 2-mercaptoethanol at a density of 20000 cells/ml and drops of about 20 *μ*l were settled on the lid of a bacterial plate, then carefully inverted and placed on the dish containing PBS. After 2 days, the spheroidal structures were harvested and incubated in GMEM medium and 100 *μ*M 2-mercaptoethanol for additional three days, at the end of which the embryoid bodies were plated on 0.1% gelatin for further 5 days.

In order to silence EPN1 and EPN2 expression, mESCs were transfected by Lipofectamine 2000 (Thermo Fisher Scientific) with vectors containing shRNAs directly against EPN1 and EPN2 or a control plasmid (Qiagen) and, then, stable lines were selected by adding puromycin.

For neuralization, mESCs were plated at a density of 1.25 × 10^4^ cells/ml and cultured up to 11 days in N2/B27 medium. NSCs were obtained by replating 7 day neuralized neural precursors on laminin (Thermo Fisher Scientific) in Euromed-N (Euroclone) supplemented with N2 (Thermo Fisher Scientific) and 20 ng/ml of both human recombinant EGF and FGF-2 (Tebubio). NICD vector (kindly provided by Prof. Miele's lab) was transfected in mESCs by nucleofection (A030 program; Nucleofector 2D, Amaxa), and cultures were then processed for the previously described analyses.

### 2.2. Western Blotting

Cell samples were lysed in RIPA buffer, and 20 *μ*g of proteins was fractionated on 9% SDS polyacrylamide gel, blotted onto PVDF membranes using the Trans Blot Turbo apparatus (BioRad). Membranes were blocked with 5% milk/TBS-T and hybridized with primary antibodies (Supplementary Table 1) diluted in 2% milk/TBS-T O/N at 4°C. Membranes were then incubated for 1 hour at room temperature with the appropriate secondary HRP-conjugated antibodies (Supplementary Table 1) diluted in 2% milk/TBS-T. The signal was detected by the ECL Clarity system (BioRad), using an Alliance Q9 chamber (Uvitec, Cambridge, UK).

### 2.3. Transferrin and N1FC Uptake Assays

To evaluate transferrin uptake, mESCs were starved for 4 hrs in GMEM w/o FBS and then incubated for 45′ at +4°C with 10 *μ*g/ml of Alexa488-transferrin (Thermo Fisher Scientific) in binding medium (GMEM, HEPES 20 mM BSA 0.1% at pH 7.4). Cells were then incubated in binding medium for 20′ at 37°C, washed in PBS, fixed in PFA 4% for 10′ at room temperature, and analysed at the microscope. For the N1FC uptake assay [27], 500 ng/ml of N1FC (Sigma) was preclustered for 1 hr at +4°C with DyLight™ 549 anti-human Fc (1 : 200, Jackson Laboratories) in GMEM + BSA 1%. The mixture was incubated with cells for 1 hr at 37°C; then cells were washed, fixed with PFA 4% for 10′ at room temperature, and analysed at the microscope.

### 2.4. Immunofluorescence Staining

Cells were fixed in PFA 4% for 15 min at room temperature, washed with PBS, and permeabilized with Triton 0.3% for 10 min at room temperature. After two brief washes, cells were incubated in blocking solution (FBS 5%, Triton 0.3% in PBS) for 1 hr and then hybridized with the primary antibodies (Supplementary Table 1) at +4°C in FBS 2% and Triton 0.2%/PBS. The signal was revealed by the appropriate secondary antibodies (Supplementary Table 1). Nuclei were counterstained with Hoechst 33258 1 *μ*g/ml (Thermo Fisher Scientific). Images were acquired with an inverted epifluorescence microscope (Leica DM IL Led Fluo with a Leica DFC450 C camera, Leica Microsystems).

### 2.5. RNA Isolation and Quantitative PCR (qPCR)

RNA was extracted with the TRIzol Reagent (Thermo Fisher Scientific) according to the manufacturer's protocol, and cDNAs were generated by the iScript cDNA Synthesis Kit (BioRad). qPCR was performed using the SsoAdvanced Universal SYBR Green Supermix Kit, with specific primers (specific sequences are reported in Supplementary Table 2). All reactions were performed in triplicate, and data were analysed according to the comparative ΔΔCt method and normalized on GAPDH or alpha-actin housekeeping genes.

### 2.6. Rosette Lumen Evaluation

To evaluate the rosette lumen diameter, neuralized mESCs were immunostained with either anti-N-cadherin or anti-ZO-1 antibodies as described in the immunofluorescence staining protocol. Rosette lumens were manually measured using the ImageJ line selection tool on calibrated images. A minimum of 80 rosettes per group were considered for the analysis.

### 2.7. Statistical Analysis

For all experiments, data are expressed as the mean ± standard deviation. The statistical significance was determined by an unpaired *t*-test using Microsoft Excel. A *P* value of less than 0.05 was considered statistically significant.

## 3. Results

### 3.1. EPN1 And EPN2 Knockdown Impairs Notch Signaling in Mouse ESCs

**Figure 1:**
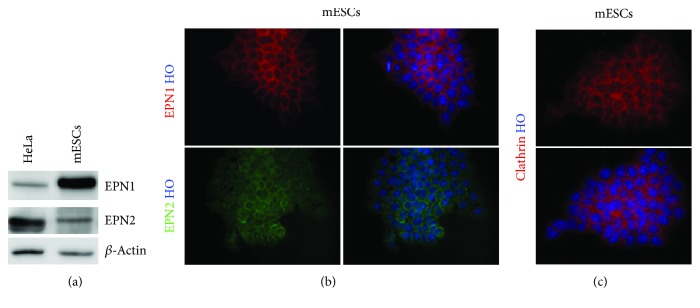
EPN1 and EPN2 are expressed in mESCs. (a) mESCs express EPN1 and EPN2. Western blot analysis shows EPN1 and EPN2 expression in mESCs. *β*-Actin was used as the loading control. (b, c) Representative immunofluorescence images of (b) EPN1 and EPN2 and (c) clathrin (heavy chain) localization in mESC: both EPNs and clathrin are localized as punctuated dots at the plasma membrane and in the cytoplasm, a pattern typical of endocytic proteins. Nuclei are counterstained with Hoechst 33258. For (a, b, and c), *n* = 3 biologically independent experiments.

EPN1 and EPN2 have been shown to be ubiquitously expressed in the mouse embryo during early phases of development [17]. Based on these data, we investigated the expression levels and potential endocytic role of epsins in mouse ESCs (mESCs). We found that EPN1 and EPN2 were abundantly expressed in mESCs (Figure 1(a)), with a characteristic punctate staining both at the plasmalemma and in the cytoplasm, with a pattern highly reminiscent of endocytic proteins (Figure 1(b)); for example, the essential endocytic coat component clathrin was found to have a similar distribution in mESCs (Figure 1(c)). Expression analysis of EPN1 and EPN2 confirmed their ubiquitous presence in different mESC lines (Supplementary Figure 1A).

EPNs have been reported to be important regulators of Notch signaling by their action on Notch ligands [21]. Both Notch ligands and Notch 1 are expressed in the different ESC lines analysed (Supplementary Figures 1B-1C).

**Figure 2:**
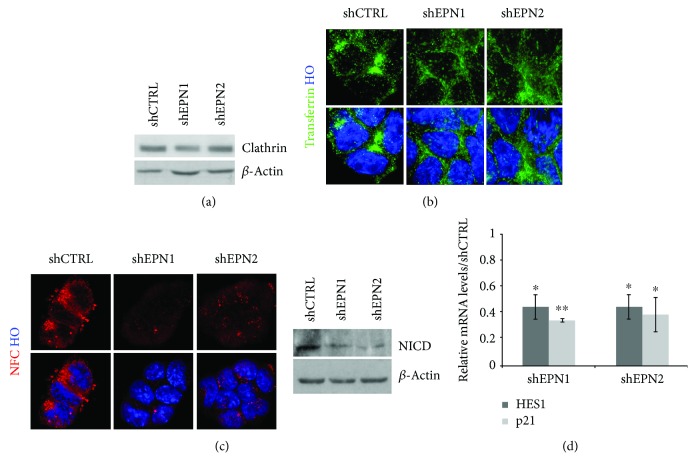
EPN1 and EPN2 silencing in mESCs impairs Notch pathway activation. (a) EPN1 and EPN2 KD does not affect clathrin levels. Western blot assay shows comparable clathrin levels in shCTRL and shEPN cells. *β*-Actin was used as the loading control. (b) EPN KD does not affect clathrin-mediated endocytosis. Representative images of clathrin-mediated uptake of Alexa488-transferrin in shCTRL and EPN1 and EPN2 KD cultures. Nuclei are counterstained with Hoechst 33258. (c) EPN KD impairs Notch activation. EPN KD abolishes uptake of N1FC and reduces NICD levels. Nuclei are counterstained with Hoechst 33258. *β*-Actin was used as the loading control. (d) EPN1 and EPN2 KD result in reduced expression levels of Notch-regulated target genes. qRT-PCR analysis of HES1 and p21 in shEPN1 and shEPN2 KD mESCs. Levels are normalized over the control line (shCTRL), using *β*-actin as the housekeeping gene. All data are expressed as the means ± STDV (*n* = 3 biologically independent experiments). Statistical significance (unpaired *t*-test): ^∗^ *p* < 0.05 and ^∗∗^ *p* < 0.001.

To investigate a possible role of single epsins in housekeeping endocytosis in mESCs, we undertook transferrin uptake assays in CTRL versus EPN knockdown (KD) mESCs. As a first step, we generated mESCs in which control shRNA (shCTRL), EPN1-shRNA (shEPN1), and EPN2-shRNA (shEPN2) were stably expressed. EPN1/2-shRNAs induced a consistent reduction of EPN expression at both the mRNA and protein levels (Supplementary Figure 2A). Overall, EPN KD mESCs did not exhibit significant phenotypical changes (Supplementary Figure 2B) in terms of cell survival, proliferation rate, and induction of differentiation (not shown); furthermore, EPN KD did not induce impairments in clathrin expression (Figure 2(a)) and localization (not shown).

Then, we measured the efficiency of transferrin uptake and we did not score any significant alteration in transferrin internalization between CTRL and EPN KD cells (Figure 2(b)); similar results were also observed in acute shEPN KD experiments. These data confirm that single EPN1 and EPN2 KD does not impair housekeeping endocytosis in mESCs.

As a consequent step, we assayed the internalization of N1FC—a recombinant soluble form of Notch-1 that is used to test the integrity of Notch ligand internalization—in mESCs in which EPN1 and EPN2 were stably knocked down. As a result of single EPN interference, we observed a dramatic reduction in the internalization of N1FC, with a concomitant decrease in NICD (Figure 2(c)), the active form of the Notch receptors for signaling. To verify that the soluble N1Fc is effectively binding to Notch ligands and prevents their interaction with Notch receptors, we assayed the downregulation of Notch primary (Hes1) and secondary (p21) target gene expressions (Figure 2(d)).

Taken together, our results strongly suggest that EPN1 and EPN2 do not exert an essential function in housekeeping endocytosis in mESCs, but they are rather required for a specialized endocytic process pivotal for the ligand-dependent activation of the Notch signaling pathway.

### 3.2. Pluripotency Maintenance in mESCs Is Altered following EPN1 and EPN2 Silencing

**Figure 3:**
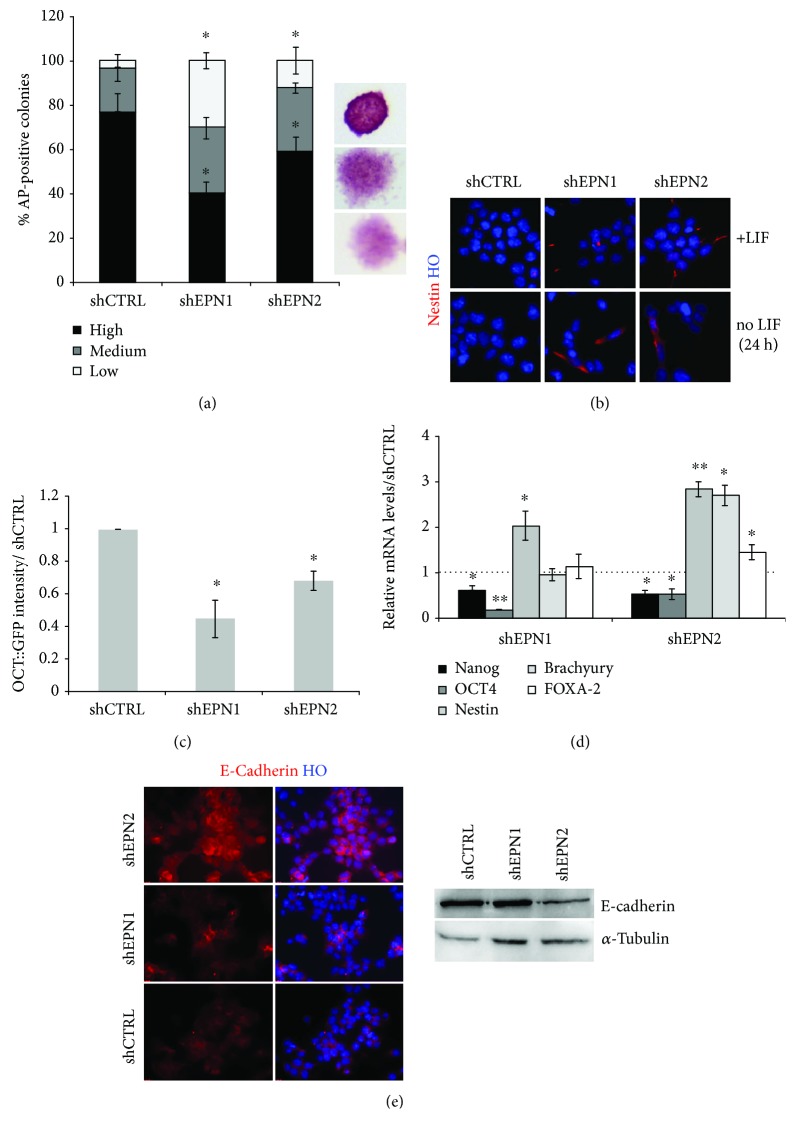
EPN1 and EPN2 KD affects pluripotency and stimulates mESC differentiation. (a) EPN1 and EPN2 silencing in mESCs impairs colony formation capability. Colony formation assays show that EPN1 and EPN2 silencing results in an increase of colonies with low alkaline phosphatase activity and a flat, differentiated morphology. Values are normalized over the control line (shCTRL). (b) EPN1 and EPN2 silencing in mESCs induces nestin upregulation upon LIF withdrawal. Representative immunofluorescence analysis for nestin expression on self-renewing or LIF-deprived EPN1 and EPN2 KD mESCs. Nuclei are counterstained with Hoechst 33258. (c) EPN1 and EPN2 KD results in a significant reduction of OCT4^+ve^ cells after 72 hours of LIF withdrawal. Values are normalized over the control line (shCTRL). (d) qRT-PCR analysis showing relative expression of pluripotency (Nanog and OCT4), neuroectodermal (nestin), mesodermal (Brachyury), and endodermal (FOXA-2) markers in EPNs silenced 46C mESCs. Data are normalized over the control line (shCTRL), using *β*-actin as the housekeeping gene. (e) Representative immunofluorescence and western blot analyses show the decrease of E-cadherin in EPN1 and EPN2 KD mESCs. *α*-Tubulin is used as the loading control, and nuclei are counterstained with Hoechst 33258. All data are expressed as the means ± STDV (*n* = 3 biologically independent experiments). Statistical significance (unpaired *t*-test): ^∗^ *p* < 0.05 and ^∗∗^ *p* < 0.001.

Notch signaling has been shown to play a key role in regulating mESC self-renewal and fate choice [28]. Hence, we investigated whether alterations in Notch signaling triggered by EPNs silencing might impact pluripotency. Colony formation assays and AP staining indicated a similar efficiency in colony formation capability between shEPN1/2 and shCTRL cells. We scored a slight reduction (about 25%) in the colony number in shEPN1 mESCs, while this parameter was unaffected in EPN2 KD cells (data not shown). Notably, the overall colonies' quality in terms of morphology and AP staining intensity was strongly affected in shEPN1/2 cells (Figure 3(a)). Indeed, the frequency of high-quality colonies (compact morphology, high AP staining intensity) was reduced in shEPN cells (40.10% and 58.81% for shEPN1 and shEPN2, respectively; shCTRL cultures: 76.61%) with a concurrent increase in medium-quality (compact morphology, intermediate AP staining intensity; 29.54% and 28.82% for shEPN1 and shEPN2, respectively; shCTRL cultures: 19.99%) and low-quality colonies (spread morphology, low AP staining intensity; 30.36% and 12.37% for shEPN1 and shEPN2, respectively; shCTRL cultures: 3.40%) (Figure 3(a)). These results indicate that EPNs might exert a role in favouring pluripotency maintenance in mESCs.

To test whether EPN1 and EPN2 KD cells have a higher tendency to exit pluripotency, we challenged the cultures in LIF-deprived medium for 24 hours, a condition known to alter mESC pluripotent state, promoting cellular differentiation [29, 30]. In these conditions, we detected an increased appearance of differentiated cells. In particular, a three-fold and two-fold increase was observed in the number of nestin^+ve^ cells in EPN1 and EPN2 KD cells, respectively (Figure 3(b)). Also, after 72 hours in these conditions, we assessed a three-fold and two-fold reduction in the percentage of OCT4^+ve^ cells in EPN1 and EPN2 KD cultures, respectively (Figure 3(c)).

To further validate the increased tendency of EPN1 and EPN2 KD cells to exit pluripotency, the expression of markers of the three germ layers derivatives was assayed by qPCR assay after 24 hours of LIF deprivation (Figure 3(d)). This analysis showed that EPN1 and EPN2 KD determined a marked decrease of Nanog (of about 50%) and OCT4 (of about 80% and 50%, respectively) transcripts with a concomitant elevation of the neuroectoderm marker nestin (two-fold induction in EPN1 KD cells and three-fold induction in EPN2 KD cells). Notably, EPN2 KD cells exhibited an additional upregulation of mesoderm (Brachyury) and endoderm (FOXA-2) markers.

To further consolidate these data in a different mESC line, we performed shEPN1/2 KD in OG2 mESCs, a reporter line in which GFP is under the control of the OCT4 promoter [26]. Interfered OG2 mESCs exhibited a marked reduction of EPN1 and EPN2 at both the transcript (not shown) and protein level (Supplementary Figure 3A), coupled to an impairment in Notch signaling activation (Supplementary Figure 3B). Analysis of GFP fluorescence after 48 hours of LIF deprivation enlightened a reduction of the OCT4^+ve^ cells in response to EPN1 and EPN2 KD (Supplementary Figure 3C). In particular, we found a 54.74% and 31.59% reduction of the overall GFP fluorescence in EPN1 and EPN2 KD cells versus control cultures, respectively (Supplementary Figure 3D).

Since several studies have reported a close connection between Notch and E-cadherin [31–33], a membrane-spanning protein of the adherent junctions essential for embryonic development and pluripotency maintenance [34–36], we decided to explore if EPN1 and EPN2 KD, associated to the impairment of Notch receptor trans-endocytosis, could impact on E-cadherin expression. We found that EPN KD resulted in a strong decrease of total E-cadherin levels (levels of E-cadherin expression with respect to the control: 54.51% and 73.12% in shEPN1 and shEPN2 KD cells, respectively) (Figure 3(e)).

In summary, our data show that downregulation of EPN1 and EPN2 levels induces early loss of pluripotency and accelerated differentiation of mESCs.

### 3.3. EPN1 and EPN2 Silencing Affects Differentiation Capability of mESCs

Considering that EPN1 and EPN2 KD hampers pluripotency maintenance, we further explored the consequences of this behaviour in differentiating conditions. Toward this aim, we first analysed the trilineage commitment competence of EPN1 and EPN2 KD mESCs by the embryoid body (EB) formation assay. No differences in the EB number or size were detected at day 5 of differentiation (data not shown). Nonetheless, qPCR analysis for lineage-specific markers revealed that EBs derived from EPN1 KD mESCs exhibited higher nestin levels if compared to the control (Supplementary Figure 4A), indicating an increased competence and/or promptness to acquire a neuroectodermal fate, as already shown in self-renewal conditions (Figure 3(d)). Notably, EBs derived from EPN2 KD mESCs exhibited a general upregulation of meso-, endo-, and ectodermal markers (Supplementary Figure 4A).

After 5 additional days of adhesion culture on gelatine, EBs derived from EPN1 and EPN2 KD mESCs exhibited a marked upregulation of trilineage differentiation markers (Supplementary Figures 4B and 4C), although at this time-point, no gross differences were visible between EBs derived from EPN1 KD and EPN2 KD cells. These results possibly pointed out an enhanced efficiency and/or a reduced time required in undertaking differentiation programs in EPN1 and EPN2 KD mESCs. To dissect between these two hypotheses (i.e., enhanced differentiation efficiency vs reduced differentiation timing), we analysed the behaviour of cell cultures during the process of ESC commitment to neural progenitor cells (NPCs). mESCs can be efficiently converted into NPCs in monolayer cultures exposed to serum-free medium (SFM) conditions [24]. The timing and efficiency of this conversion can be qualitatively and quantitatively monitored by means of the SOX1::GFP reporter mESCs (46C cells [24]). Using this assay, we found that, as expected, shCTRL cells showed a gradual appearance of GFP^+ve^ cells by day 4 (Supplementary Figure 5A) and a peak at day 7. In contrast, EPN1 and EPN2 KD cells exhibited an appearance of GFP^+ve^ cells by day 3 with an anticipated peak at days 5-6 (Supplementary Figure 5C). These results indicate that EPN1 and EPN2 KD do not affect the overall neural conversion efficiency, but it rather induces an accelerated commitment to neural fate.

In order to check this hypothesis, day 7 neuralized cultures were immunostained for *β*III-tubulin, a marker of maturing neurons. EPN1 and EPN2 KD cultures exhibited an increased number of *β*III-tubulin^+ve^ cells at this stage compared to shCTRL cultures (Supplementary Figure 6A), indicating that EPN1 and EPN2 silencing induced a precocious appearance of neuronal cells. Western blot analysis confirmed the increased accumulation of neurons in day 7 EPN silenced cultures, showing also an expression of the astroglial marker GFAP in EPN2 KD cultures (Supplementary Figure 6B).

To better evaluate *β*III-tubulin accumulation during the neuralization process, we performed EPN1 and EPN2 silencing in *β*III-tubulin::eGFP cell reporter mESCs [25]. After confirming effective EPN1/2 KD (not shown), stable shCTRL and shEPN1/2 cultures were exposed to the neuralization process. Consistently, both shEPN1 KD and shEPN2 KD lines exhibited a premature and enhanced appearance of neuronal eGFP-expressing cells (Supplementary Figure 6C).

During the mESC *in vitro* neuralization process, NPCs organize themselves in polarized structures named *neural rosettes* that share several features with developing neural tube [37]. In neural rosettes, proliferating NPCs are organized radially around a central lumen, and their differentiated progeny is arranged at the periphery. It has been shown that rosette's architecture and size are primarily linked to the precise balance between self-renewal and differentiation. Since it has been shown that modulation of Notch signaling, which we found severely impaired following EPN1 and EPN2 KD in mESCs, results in alteration of neural rosette structures, we analysed their number, size, and composition in day 7 cultures.

**Figure 4:**
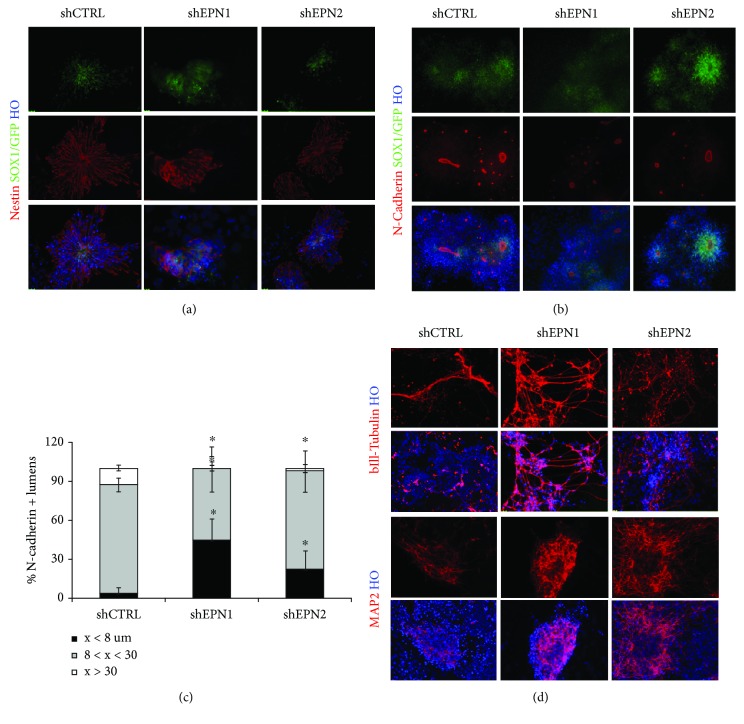
EPN1 and EPN2 silencing affects neuroepithelial cells polarization and neural rosette lumen size. (a) Nestin immunofluorescence analysis reveals impaired neuroectoderm polarization and neural rosette lumen size in 7 day-neuralized EPN1 and EPN2 KD 46C mESC cultures. (b, c) Immunofluorescence analysis and relative size quantification of N-cadherin^+ve^ lumens show that EPN1 KD and EPN2 KD induce a decrease in the percentage of large-lumen rosettes. (d) After 11 days of exposure to neuralizing conditions, EPN1 and EPN2 KD mESC cultures show an increase in *β*III-tubulin^+ve^ and MAP 2^+ve^ neurons as scored by immunofluorescence analysis. Nuclei are counterstained with Hoechst 33258. All data are expressed as the means ± STDV ((a, d): *n* = 3 biologically independent experiments; (b, c): *n* = 5 biologically independent experiments). Statistical significance (unpaired *t*-test): ^∗^ *p* < 0.05.

After 7 days of the neuralization process, shCTRL mESCs showed the presence of the expected large and well-polarized rosettes, with nestin^+ve^ cells arranged around a regular central lumen (Figure 4(a)). EPN1 KD cultures showed a comparable number of Nestin^+ve^ clusters, albeit smaller and not well-polarized. Notably, EPN2 KD determined the accumulation in the cultures of smaller nestin^+ve^ rosettes (Figure 4(d), and Supplementary Figure 6A).

Lumen size has been shown to be associated to the NPCs' ability to divide symmetrically or asymmetrically, and this feature directly correlates with the level of Notch activation [38]. In order to better define EPN1 and EPN2 contribution in NPC renewal/differentiation, the lumen diameter of the neural rosettes was assessed by N-cadherin and ZO-1 immunostaining of the cultures followed by morphometry studies (Figures 4(b) and 4(c), and Supplementary Figure 7A-7B). A strong reduction of rosette lumen size in EPN1 KD population was evident, if compared to the shCTRL cultures. Large N-cadherin^+ve^ lumens (diameter greater than 30 *μ*M) were almost completely undetectable, while about half of the total rosettes (44.12%) was characterized by small lumens (diameter lower than 8 *μ*M). EPN2-silenced cultures were characterized by a seven-fold increase of N-cadherin^+ve^ small lumens and a concomitant six-fold decrease in the percentage of large rosettes (Figure 4(c)).

Altogether, these results indicate that EPN1 and EPN2 play an important role in regulating the correct rosette polarization and in balancing NPC proliferation vs differentiation.

Finally, in order to evaluate neuronal differentiation at later stages of the neuralization process, day 11 cultures were assayed for number, morphology, and degree of neuronal maturation. Consistent with the previous results, EPN KD cells exhibited an enhanced neuronal maturation, as assessed by the appearance of an increased number of *β*III-tubulin^+ve^ and MAP 2^+ve^ neurons with mature morphological hallmarks in respect of the CTRL cultures (Figure 4(d)).

Collectively, our data show that EPN1 and EPN2 absence promotes an accelerated neuralization, although this process is severely dysregulated.

### 3.4. NICD Expression Rescues the Effects of EPN1 and EPN2 KD on the mESC Neuralization Process

Neuralized mESC cultures can be efficiently converted into homogeneous and stably self-renewing radial glia-like neural stem cell populations, named NS cells [39]. Since above results indicated that EPN1 and EPN2 KD in mESCs impairs some aspects of the neuralization process, we tested whether these cells would maintain the competence to give rise to NS cells.

Day 7 neuralized EPN KD and CTRL mESC cultures were replated onto laminin-coated plastic in permissive NS cell medium, containing EGF and FGF-2. CTRL cells promptly adapted to these conditions and efficiently gave rise to homogeneous adherent bipolar NS cell populations. In contrast, EPN1 and EPN2 KD cells gradually attached to the substrate but progressively stopped division and underwent cell death in a few days (data not shown).

**Figure 5:**
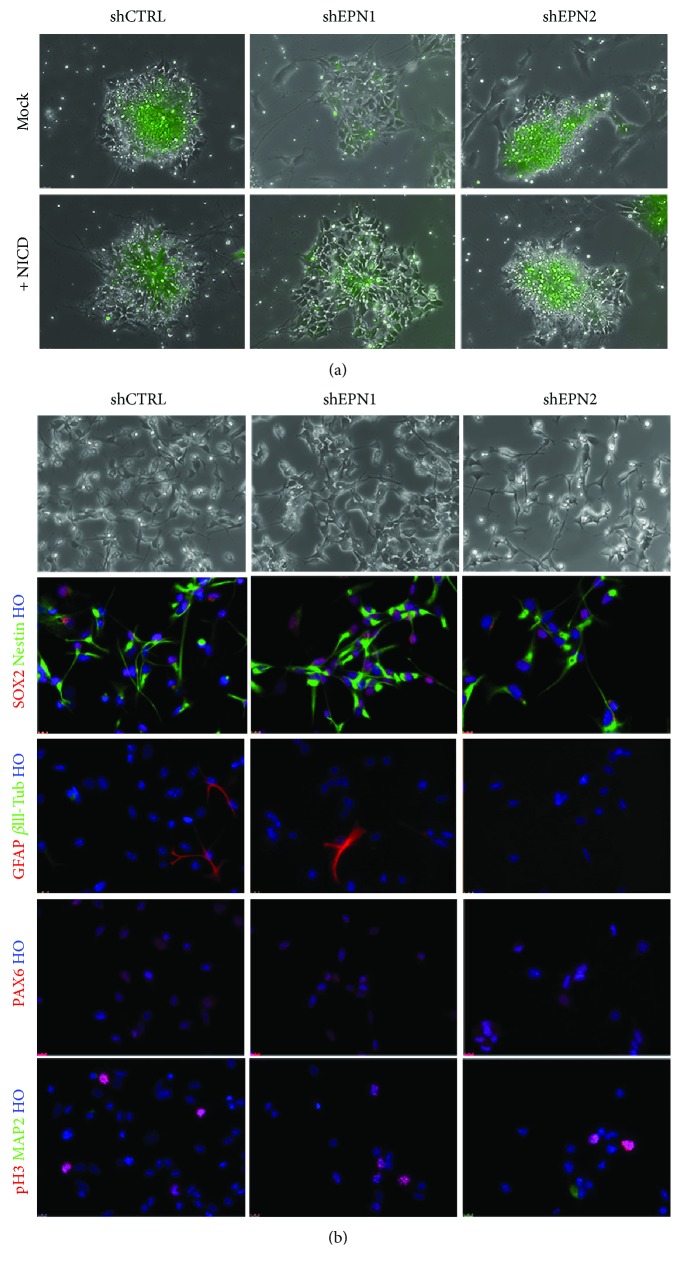
NICD overexpression rescues EPN1 and EPN2 KD phenotype. (a) NICD overexpression in EPN1 and EPN2 KD ESCs can rescue the regular and well-polarized rosette morphology during neuralization. eGFP signal indicates either mock or NICD-transfected cells. (b) Immunofluorescence analyses on mESC-derived NS cells show that NICD overexpression allows the obtainment of expandable undifferentiated EPN1 and EPN2 KD cultures (no or very few GFAP, *β*III-tubulin, and MAP 2-positive cells), homogeneously immunopositive for SOX-2, nestin, and PAX6 and with similar proliferation rate (pH 3 expression). For (a, b), *n* = 3 biologically independent experiments.

In order to evaluate if the effects of EPN1 and EPN2 silencing on the overall neuralization process can be primarily due the impairment of the EPN-mediated Notch signaling activation, we overexpressed NICD (i.e. the Notch transcriptionally active form) in shEPN and shCTRL mESCs. Cultures were then exposed to the neuralization process and tested (i) for neural rosette formation and (ii) for their competence to be converted into NS cells. After 7 days of neuralization, rosettes in the mock-transfected EPN1 and EPN2 KD cells exhibited the same phenotypes as previously described (Figure 4), with defects in structure polarity and decreased lumen size (Figure 5(a)). In contrast, EPN1 and EPN2 KD cells transfected with NICD exhibited a reestablished radial organization and lumen size (Figure 5(a)). While mock-transfected cells failed to establish a NS cell line (not shown), NICD overexpressing EPN1 and EPN2 KD cells survived and proliferated for several passages, exhibiting a WT-like phenotype (Figure 5(a)); in particular, they displayed a homogeneous expression of Nestin, PAX6, and SOX2, as well as a low index of spontaneous differentiation (Figure 5(b)). These rescue experiments confirm that the neuralization effects scored in EPN1 and EPN2 KD mESCs are indeed due to an impairment of the Notch signaling pathway, independent of possible off-target silencing artefacts.

We can conclude that Epsin1/2 knockdown leads to impaired Notch signaling activation resulting in impaired neural rosette formation and polarization and altered regulation of NS cell self-renewal and viability.

## 4. Discussion

Epsins are endocytic adaptors essential for several fundamental physiological processes in higher eukaryotes. Notably, among the others, EPNs have been shown to regulate activation of Notch signaling [7–10, 17], a pathway involved in several developmentally relevant processes. In this study, we explore the specific role played by EPNs in the biology and function of mammalian pluripotent stem cells, an issue yet unexplored in the field. We found that EPNs are expressed in mESCs and that EPN1 and EPN2 single knockdown does not affect self-renewal potential of the cells. Nevertheless, at a molecular level, downregulation of EPN1/2 expression resulted in impaired Notch activation, as scored by reduced expression of NICD, the active Notch effector, and of Hes1, a direct Notch target gene [40]. Previous studies have reported that Notch ligands and receptors are expressed in mESCs [41] and that neither activation nor inactivation of Notch signaling in these cells significantly disturbs their self-renewal potential [41–43]. It has also been shown that Hes1 levels normally undergo oscillations in mESCs and that Hes1 downregulation does not induce pluripotency exit both in *vitro* [44] and *in vivo*, since Hes1 mutants proceed successfully through gastrulation [45, 46]. Notably, Hes1 has been shown to be transiently downregulated in mESCs as they move toward differentiation into neural or nonneural lineages [46]. These results are consistent with the current view that Notch and Hes1 are not sufficient to instruct pluripotency exit *in vitro*. Instead, they play major roles in the early stages of pluripotency exit by conferring flexibility in the differentiation response through modulation of the threshold at which cells respond to differentiation signals.

In agreement with these findings, we observed that EPN1 and EPN2 silencing affects Notch signaling activation resulting in reduced high-quality colony formation in low-density plating and more rapid pluripotency marker downregulation both under general differentiation conditions (i.e., LIF deprivation and EB assays) and under neural specific differentiation conditions. Consistently, Notch activation in mESCs has been reported to potentiate LIF signaling [46] and to delay the transition toward differentiation by Hes1-mediated maintenance of STAT3 activity [47]. Here, we found that, in EB assay, EPN1 KD favours commitment to a neural fate while EPN2 silencing leads to a generalized commitment toward meso-, endo-, and ectoderm lineages. High levels of Hes1 have been reported to suppress neural induction in mESCs and neural progenitor neuronal differentiation effects that are mediated by downregulation of Notch ligands [41, 46]. Previous studies have shown that Hes1 ablation accelerates mESC differentiation and that its overexpression inhibits differentiation of ES cells into the neural linage and also delays mesoderm and endoderm differentiation [46]. We speculate that different effects produced by EPN1 and EPN2 downregulation in EB assays could be attributed to their diverse degree of interaction with the various Notch ligands. Indeed, even if the Notch pathway is highly conserved in metazoan [48], the presence of multiple ligands and receptors is not the mere expression of a functional redundancy, but rather represents a vehicle to modulate specific downstream effects [49].

A major role for Notch signaling and Hes1 has been described in a neural commitment process. Indeed, inhibition of Notch in mESCs exposed to neural differentiating conditions leads to quicker and more homogeneous commitment into neural progenitors and increased neuronal differentiation [41, 50]. Similar results are obtained when neuralizing Hes1 homozygous null mESCs. Consistently, we found that EPN1 and EPN2 silencing induces a strong accumulation of the neuronal marker *β*III-tubulin in differentiated EBs. We also confirmed that reduced Notch activation and consequent decreased Hes1 expression, due to EPN1 and EPN2 knockdown, favour neural differentiation in a neural differentiation protocol, leading to accelerated appearance and increased expression of the early neural marker SOX-1 both in EPN1- and in EPN2-silenced mESCs.

Notch signaling downregulation has been associated with defects in neural tube formation during development, negatively impacting on apical/basal cell polarity. During the *in vitro* neuralization process, there is appearance of the so-called “neural rosettes,” polarized structures that mirror *in vitro* the developing neural tube. The inhibition of Notch signaling with a genetic or pharmacological (i.e., DAPT treatment) approach has been shown to result in strong alterations in neural rosette polarity and integrity, producing also an accelerated neuronal differentiation [51]. Furthermore, previous studies have reported that Notch supports symmetrical division and expansion of neural progenitors by a HES-mediated inhibition of proneural genes expression [52–55]. We found that, similarly to Notch inhibition, EPNs silencing affects both rosette polarity and symmetrical neuroepithelial cell divisions. More specifically, EPN1 and EPN2 knockdown leads to a strong decrease in rosette lumen size and faster appearance of neuronal cells, features that are reminiscent of a precocious arrest of symmetrical division, in favour of an asymmetrical differentiating division [38]. These results point to a key role of EPNs in cell division, extending previous observations showing abnormal spindle appearance in EPN 1 and 2 double KD cells [56, 57]. To this respect, several endocytic proteins have been involved in mitosis and in cytokinesis processes [58]. During mitosis, EPNs undergo phosphorylation and ubiquitination and these covalent modifications impair their binding to clathrin and AP-2, suggesting a switch of their function in the mitotic cytosol [2, 12, 59, 60].

Finally, we showed that EPN1 and EPN2 silencing completely prejudices the generation of stable self-renewing radial glia-like NS cells [39, 61–63]. Indeed, EPN1 and EPN2 knockdown affects NS cell proliferation by forcing them to prematurely differentiate and undergo cell death. These effects are Notch-dependent since overexpression of the activated form of Notch1 completely rescues these aberrant phenotypes. This role of EPNs in NSC self-renewal and maintenance is in agreement with previous studies enlightening the importance of endocytic machinery-mediated Notch activation in NSC behaviour in *Drosophila*. Indeed, mutants in *α*-adaptin gene, a member of the clathrin adaptor AP-2 complex, exhibited an anomalous Notch trafficking, responsible for the misregulated neuronal fate acquisition in the fly [64].

## 5. Conclusions

This study set out to determine the action played by EPNs and endocytosis in mESC biology. Our data show that EPNs are expressed in mESCs and that they contribute to control Notch signaling activation thus regulating pluripotency exit and lineage commitment processes. Taken together, these results disclose new aspects of the EPN function in mESCs and point to endocytosis as an important contributor of the dynamics of early developmental-regulated processes.

## Data Availability

The data used to support the findings of this study are available from the corresponding author upon request.

## Supplementary Materials

Supplementary Materials Supplementary Figure 1: western blot and qRT-PCR analyses for detection of expression levels of EPNs and Notch ligands in mESC lines. Supplementary Figure 2: western blot and qRT-PCR analyses for detection of EPN1 and EPN2 levels in stable KD mESC lines. Phase contrast images of EPN1 and EPN2 stable KD mESC lines. Supplementary Figure 3: western blot that shows the efficient EPN1 and EPN2 silencing in OG2 mESCs. Representative immunofluorescence images for NCID immunoreactivity in shCTRL and shEPN/2 KD cells. Representative immunofluorescence images for OCT4::GFP and chart that show the quantification of OCT4::GFP fluorescence levels in shCTRL and shEPN cells (values are normalized over the shCTRL line). Supplementary Figure 4: qRT-PCR analysis that shows relative expression of differentiation markers in EB assay cultures (results are normalized over the shCTRL line). Representative immunofluorescence images and western blot analysis for germ layer differentiation markers scored on 10 day differentiated EPN1 and EPN2 KD EB cultures. Supplementary Figure 5: representative images of SOX1::GFP^+ve^ cell clusters for shCTRL and EPN1- and EPN2-silenced 46C mESC cultures. Chart shows quantification of SOX1::GFP^+ve^ cells in control and EPN1 and EPN2 KD cultures at 4 days of neuralization process. Evaluation of SOX1::GFP fluorescence in EPN1 and EPN2 KD cultures at different time points of the neuralization process. Supplementary Figure 6: *β*III-tubulin immunostaining performed on SOX1::GFP 46C mESC cultures at 7 days of the neuralization process. Western blot that shows neuronal and astroglial marker expression levels in EPN1 and EPN2 KD and control cultures following 7 days of exposure to neuralizing conditions. Representative pictures show eGFP expression in control and EPN1 and EPN2 KD *β*III-tubulin::eGFP mESC cultures at 6 and 7 days of neuralization. Supplementary Figure 7: ZO-1 immunostaining on 7 day neuralized shEPN1 46C mESC cultures. Chart reports the quantification of rosette's lumen diameters in 7 day neuralized shEPN1/shEPN2 and shCTRL mESC cultures. Supplementary Table 1: list of antibodies used in this study. Supplementary Table 2: list of primers (and relative sequences) used in this study.
