# Supplementary material for: Epsins Regulate Mouse Embryonic Stem Cell Exit from Pluripotency and Neural Commitment by Controlling Notch Activation
Source: Stem Cells Int. 2019 Feb 25;2019:4084351. doi: 10.1155/2019/4084351 (PMC6410434; doi:10.1155/2019/4084351)
Supplement: Supplementary Materials — Supplementary Figure 1: western blot and qRT-PCR analyses for detection of expression levels of EPNs and Notch ligands in mESC lines. Supplementary Figure 2: western blot and qRT-PCR analyses for detection of EPN1 and EPN2 levels in stable KD mESC lines. Phase contrast images of EPN1 and EPN2 stable KD mESC lines. Supplementary Figure 3: western blot that shows the efficient EPN1 and EPN2 silencing in OG2 mESCs. Representative immunofluorescence images for NCID immunoreactivity in shCTRL and shEPN/2 KD cells. Representative immunofluorescence images for OCT4::GFP and chart that show the quantification of OCT4::GFP fluorescence levels in shCTRL and shEPN cells (values are normalized over the shCTRL line). Supplementary Figure 4: qRT-PCR analysis that shows relative expression of differentiation markers in EB assay cultures (results are normalized over the shCTRL line). Representative immunofluorescence images and western blot analysis for germ layer differentiation markers scored on 10 day differentiated EPN1 and EPN2 KD EB cultures. Supplementary Figure 5: representative images of SOX1::GFP+ve cell clusters for shCTRL and EPN1- and EPN2-silenced 46C mESC cultures. Chart shows quantification of SOX1::GFP+ve cells in control and EPN1 and EPN2 KD cultures at 4 days of neuralization process. Evaluation of SOX1::GFP fluorescence in EPN1 and EPN2 KD cultures at different time points of the neuralization process. Supplementary Figure 6: βIII-tubulin immunostaining performed on SOX1::GFP 46C mESC cultures at 7 days of the neuralization process. Western blot that shows neuronal and astroglial marker expression levels in EPN1 and EPN2 KD and control cultures following 7 days of exposure to neuralizing conditions. Representative pictures show eGFP expression in control and EPN1 and EPN2 KD βIII-tubulin::eGFP mESC cultures at 6 and 7 days of neuralization. Supplementary Figure 7: ZO-1 immunostaining on 7 day neuralized shEPN1 46C mESC cultures. Chart reports the quantification [file 4084351.f1.docx]

**SUPPLEMENTARY MATERIALS**

**Supplementary Table 1.** Antibodies List

| Antibody | Company | Code | Dilution |
| --- | --- | --- | --- |
| anti-CK8 | Santa Cruz Biotechnology | sc-101459 | 1:1000 (WB); 1:100 (IF) |
| anti-Clathrin Heavy Chain | BD Biosciences | 610500 | 1000 (WB); 1:50 (IF) |
| anti-E-Cadherin | Santa Cruz Biotechnology | sc-7870 | 1:500 (WB); 1:100 (IF) |
| anti-EPN1 | Cogentech; | 42R2 | 1:30 (WB, IF) |
| anti-EPN2 | Santa Cruz Biotechnology | sc-5414 | 1:1000 (WB); 1:50 (IF) |
| anti-GFAP | DAKO | Z0334 | 1:1000 (WB); 1:500 (IF) |
| anti-MAP-2 | BD Biosciences | 556320 | 1:200 (IF) |
| anti-N-Cadherin | Santa Cruz Biotechnology | sc-7939 | 1:100 (IF) |
| anti-Nestin | Santa Cruz Biotechnology | sc-33677 | 1:1000 (WB); 1:200 (IF) |
| anti-NICD | Cell Signalling | 2421s | 1:500 (IF) |
| anti-Notch-1 | Santa Cruz Biotechnology | sc-373891 | 1:1000 (WB); 1:100 (IF) |
| anti-PAX6 | Santa Cruz Biotechnology | sc-11357 | 1:100 (IF) |
| anti-pH3 | Millipore; | 04-1093 | 1:100 (IF) |
| anti-SOX-17 | Santa Cruz Biotechnology | sc-17356 | 1:1000 (WB) |
| anti-SOX2 | Millipore; | AB5603 | 1:300 (IF) |
| anti-ZO1 | Thermo Fisher Scientific | PAD: Z-R1 | 1:100 (IF) |
| anti-α-Actin | SIGMA | A2228 | 1:2000 (WB) |
| anti-α-SMA | SIGMA | A 2547 | 1:2000 (WB); 1:1000 (IF) |
| anti-αTubulin | Santa Cruz Biotechnology | sc-53646 | 1:1000 (WB) |
| anti-βIII-Tubulin | Promega | G712A | 1:1000 (WB); 1:1000 (IF) |
| Donkey anti-goat IgG-HRP | Santa Cruz Biotechnology | sc-2020 | 1:500 (WB) |
| Donkey anti-Goat IgG, Alexa Fluor 488 | Thermo Fisher Scientific | A11055 | 1:400 (IF) |
| Goat anti-mouse IgG-HRP | Biorad | 1705047 | 1:3000 (WB) |
| Goat anti-Mouse IgG, Alexa Fluor 488 | Thermo Fisher Scientific | A11001 | 1:400 (IF) |
| Goat anti-Mouse IgG, Alexa Fluor 568 | Thermo Fisher Scientific | A11004 | 1:400 (IF) |
| Goat anti-rabbit IgG-HRP | Biorad | 1705046 | 1:3000 (WB) |
| Goat anti-Rabbit IgG, Alexa Fluor 488 | Thermo Fisher Scientific | A11008 | 1:400 (IF) |
| Goat anti-Rabbit IgG, Alexa Fluor 568 | Thermo Fisher Scientific | A11011 | 1:400 (IF) |

**Supplementary Table 2.** Primers list

| **Gene** | **Primer sequence** | **Product lenght (bp)** |
| --- | --- | --- |
| **Brachyury** | F- CCAGCTCTAAGGAACCACCG | 109 |
|  | R- ACTGCAGCATGGACAGACAA |  |
| **Dll1** | F- AACACTTAGGGGTGGGGAGA | 154 |
|  | R- TGCCACATCGCTTCCATCTT |  |
| **Epn1** | F- GACTTCGACCGACTCCGCAC | 124 |
|  | R- ATCCCCCAACCCCACTCATGTC |  |
| **Epn2** | F- AAGAAAGCCGGAGAGACAC | 156 |
|  | R- CACTCCAGGGCTCAGTTTTC |  |
| **Foxa2** | F- GGCCCAGTCACGAACAAAGC | 177 |
|  | R- CCCAAAGTCTCCACTCAGCCTC |  |
| **Hes1** | F- CCAGCCAGTGTCAACACGA | 166 |
|  | R- AATGCCGGGAGCTATCTTTCT |  |
| **Jag1** | F- CCTGCGAGCCAAGGTGTG | 104 |
|  | R- CTCCACCACAACAGTTCCCA |  |
| **Jag2** | F- CTGTGCGCTCGACATTGATGA | 165 |
|  | R- AAAGCATTAAGGCACGGCTTC |  |
| **Nanog** | F- AGTATCCCAGCATCCATTGC | 186 |
|  | R- AGTATCCCAGCATCCATTGC |  |
| **Nestin** | F- CATACAGGACTCTGCTGGAGG | 130 |
|  | R- AGGTGCTGGTCCTCTGGTAT |  |
| **Oct4** | F- GGAGAAGTGGGTGGAGGAA | 188 |
|  | R- GCTGATTGGCGATGTGAG |  |
| **p21** | F- CTGGTTCCTTGCCACTTCTT | 131 |
|  | R- GGCTGTGACTGCTTCACTGT |  |

**
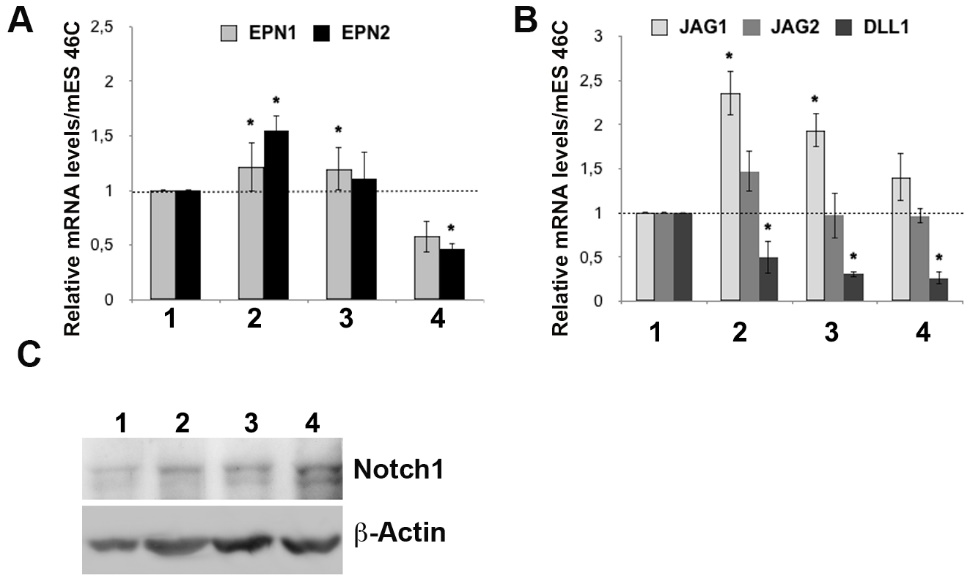
**

**Supplementary figure 1.** **EPNs and Notch ligands are expressed in mESC lines**.

**(a)** EPN1/2 and **(b)** Notch ligands are expressed in self-renewing mESCs. qRT-PCR analyses for EPN1/2 and Notch ligands expression in different mESC lines (1: 46C; 2: E14; 3: βIII-Tubulin::eGFP; 4: OG2). Transcript levels are normalized over a 46C mESCs, using GAPDH as housekeeping gene. **(c)** Notch1 protein is expressed in self-renewing mESCs. Western blot analysis for detection of Notch1 receptor levels in different mESCs lines. β-Actin was used as loading control. All data are expressed as the means ± STDV (n = 3 biologically independent samples). Statistical significance (unpaired t-test): *p < 0.05, **p < 0.001.


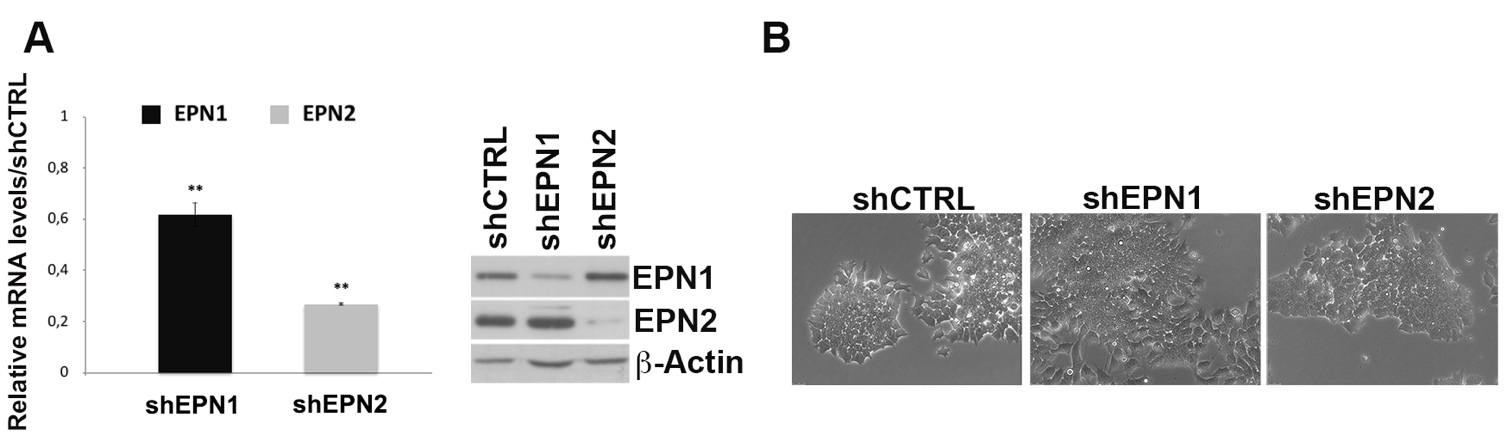


**Supplementary figure 2.** **EPN1 and EPN2 stable KD does not affect mESCs phenotype.**

**(A)** Efficient EPN1 and EPN2 KD in mESCs. qRT-PCR (left) and western blot (right) analyses for EPN1 and EPN2 expression in EPNs stable KD mESC lines. EPN1/2 transcripts levels are normalized over the control line (shCTRL), using β-Actin as loading control. **(B)** EPN1 and EPN2 KD do not induce visible phenotypic differences in mESC cultures. Representative phase contrast images of self-renewing CTRL and EPNs KD mESC cultures. All data are expressed as the means ± STDV (n = 3 biologically independent experiments). Statistical significance (unpaired t-test): *p < 0.05, **p < 0.001.


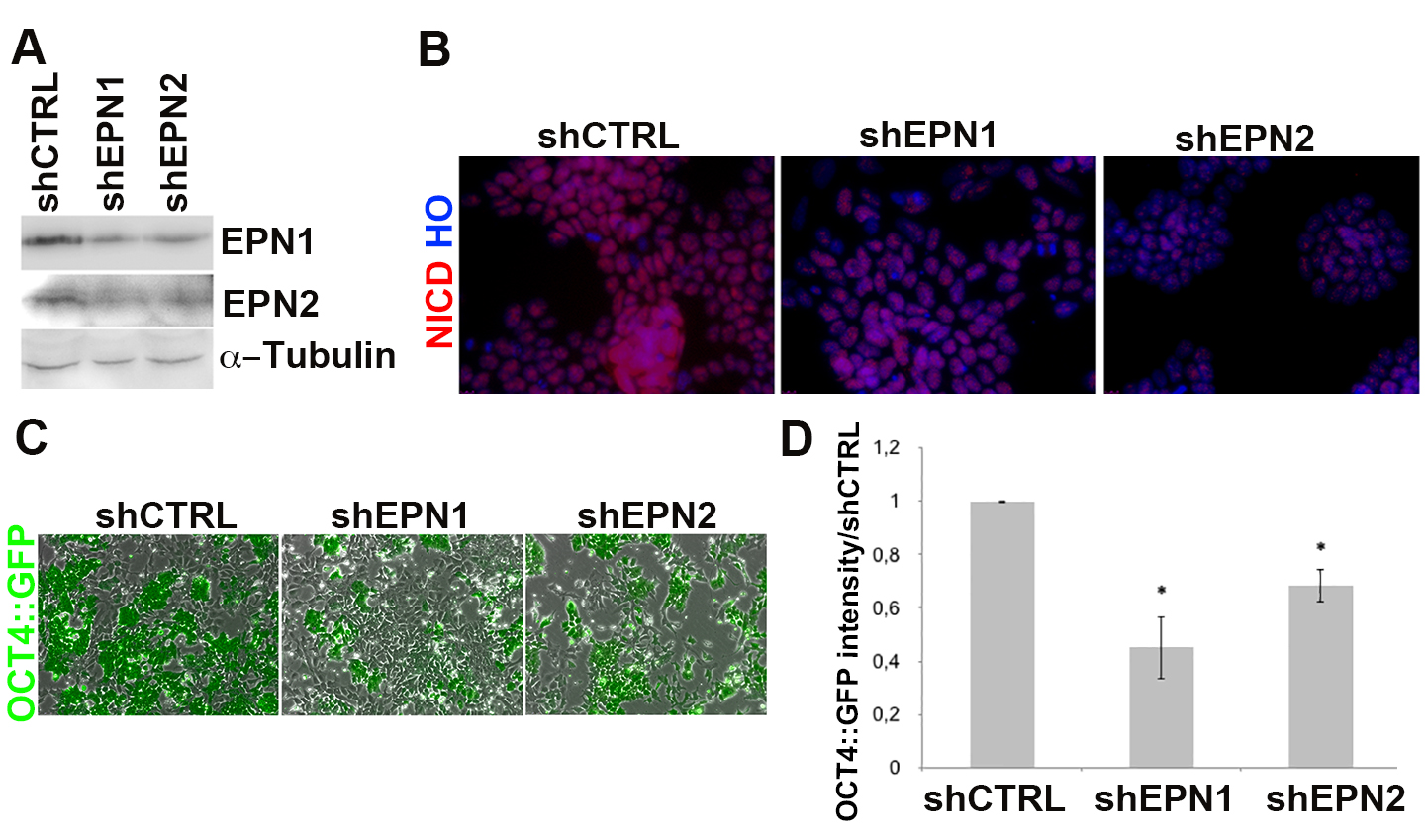


**Supplementary figure 3.** **EPN1 and EPN2 KD impairs Notch signalling and OCT4 expression in OG2 mESCs.**

**(A)** shRNA-mediated efficient EPN1 and EPN2 KD in OG2 mESCs. Western blot indicating the efficient EPN1 and EPN2 silencing in OG2 mESCs. α-Tubulin was used as loading control. **(B)** EPN1 and EPN2 KD in OG2 mESCs impairs Notch pathway activation. Representative immunofluorescence images for NCID immunoreactivity in shCTRL and shEPNs cells. Nuclei are counterstained with Hoechst 33258 **(C-D)** EPN1 and EPN2 KD affects expression levels of OCT4::GFP. OCT4::GFP fluorescence levels are normalized over the control line (shCTRL). All data are expressed as the means ± STDV (n = 3 biologically independent experiments). Statistical significance (unpaired t-test): *p < 0.05.


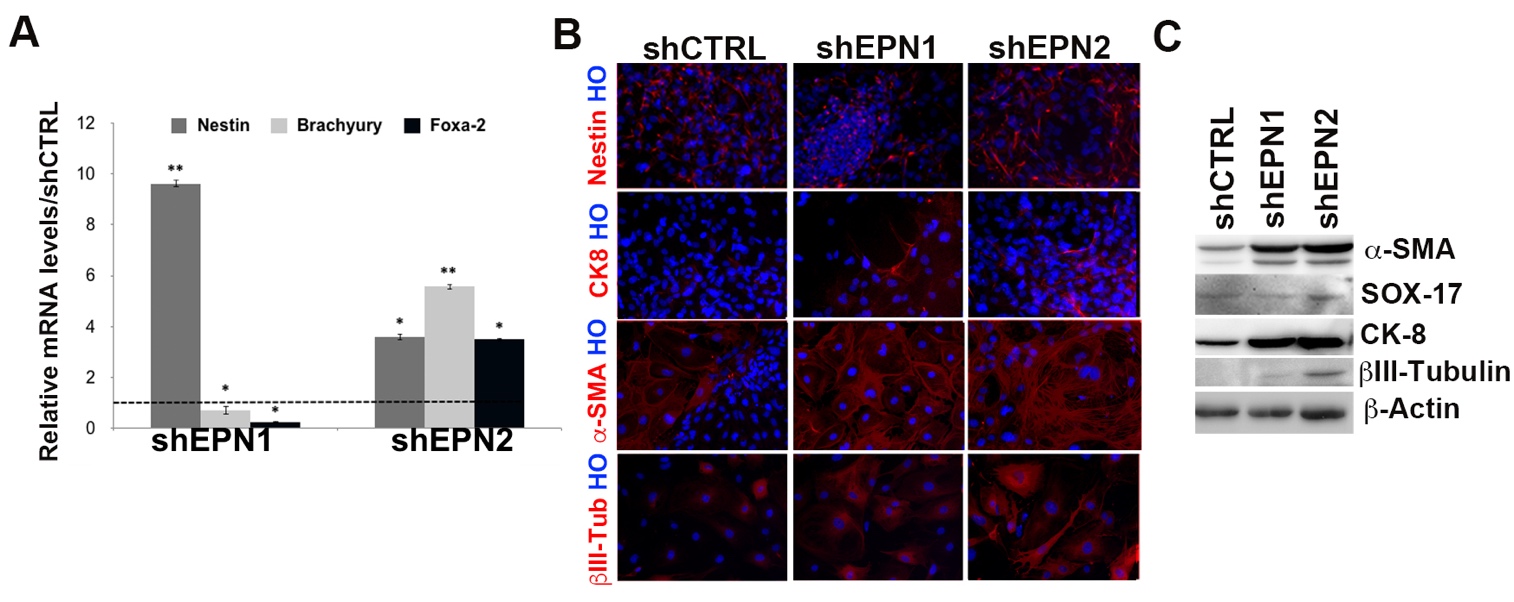


**Supplementary figure 4.** **EPN1 and EPN2 KD promote mESCs differentiation during EBs formation.**

(A) qRT-PCR analysis showing relative expression of differentiation markers in EB assay cultures derived from EPN1 and EPN2 KD 46C mESCs. Results are normalized over control line (shCTRL), using β-Actin as housekeeping gene. (B and C): Representative immunofluorescence images and western blot analysis for germ layers differentiation markers scored on 10 days differentiated EPNs KD EB assay cultures. Nuclei are counterstained with Hoechst 33258; β-Actin is uses as loading control. All data are expressed as the means ± STDV (n = 3 biologically independent experiments). Statistical significance (unpaired t-test): *p < 0.05, **p<0.001.


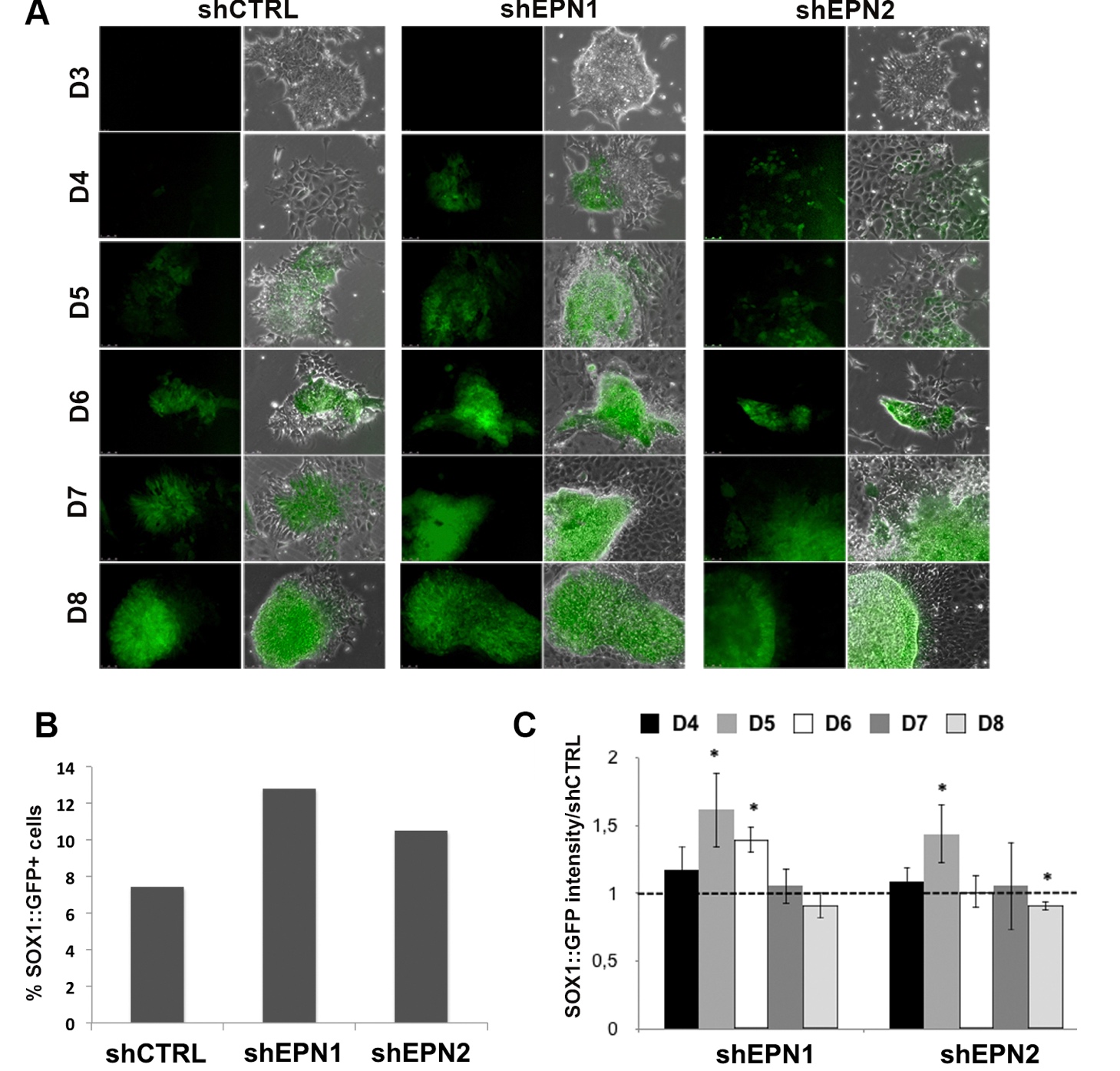


**Supplementary figure 5.** **EPN1 and EPN2 KD accelerate SOX1 expression during neuralization process.**

**(A)** EPN1 and EPN2-silenced 46C mESC cultures exhibit an accelerated appearance of SOX1::GFP+ve cell clusters. **(B)** Tali™ Image-Based Cytometer quantification of SOX1::GFP^+ve^ cells in control and EPN1 and EPN2 KD cultures at 4 days of neuralization process. **(C)** Evaluation of SOX1::GFP fluorescence in EPN1 and EPN2 KD cultures at different time points of the neuralization process,. Data are normalized over the time-matched shCTRL cells values. All data are expressed as the means ± STDV (n = 3 biologically independent experiments). Statistical significance (unpaired t-test): *p < 0.05.


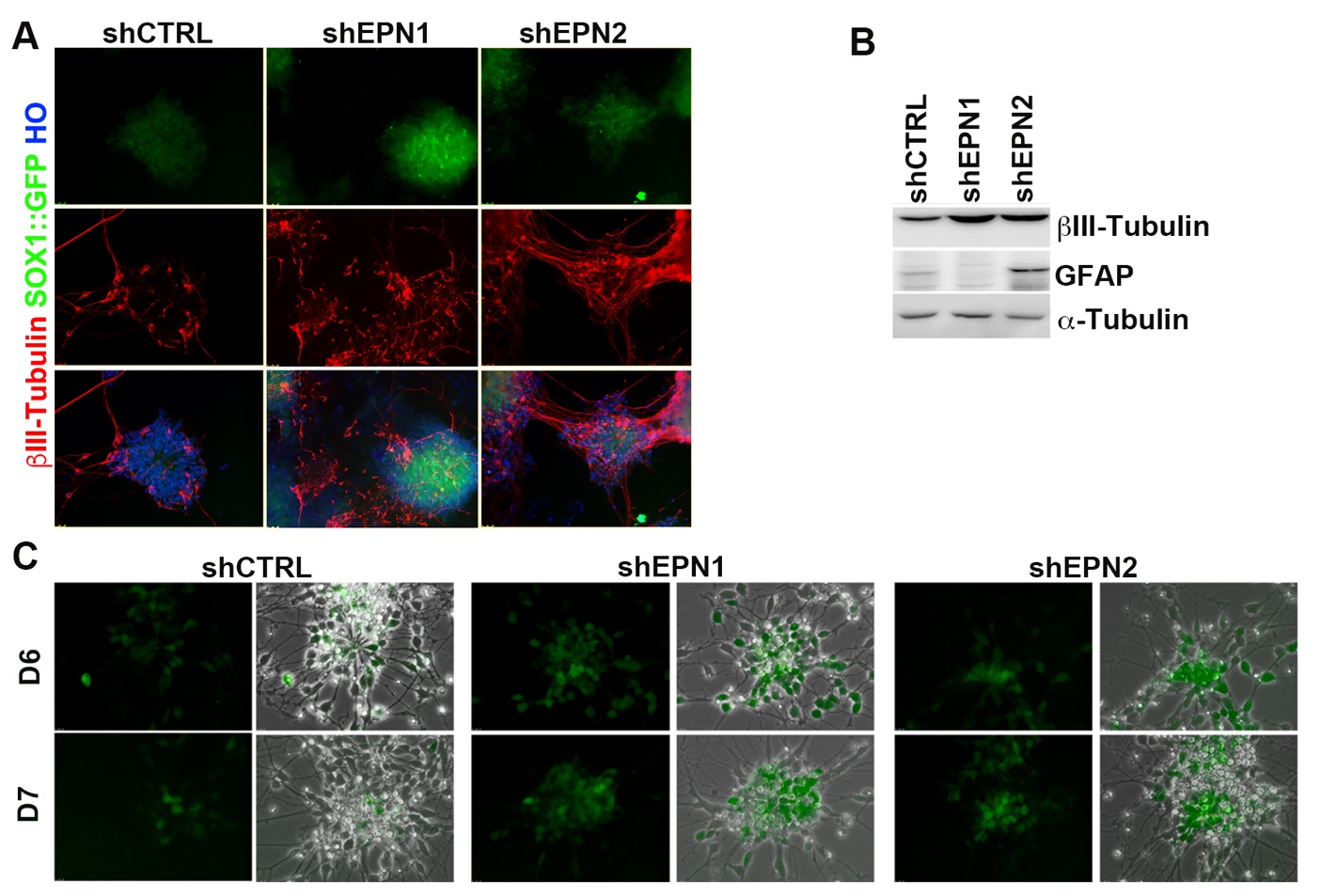


**Supplementary figure 6.** **EPN1 and EPN2 KD favour mESCs neuronal differentiation.**

(A) βIII-Tubulin immunostaining performed on SOX1::GFP 46C mESCs cultures at 7 days of neuralization process. Nuclei are counterstained with Hoechst 33258. (B) Western blot assessing neuronal (βIII-Tubulin) and astroglial (GFAP) markers expression levels in EPN1 and EPN2 KD and control cultures following 7 days of exposure to neuralizing conditions. α-Tubulin is used as loading control (representative picture of n = 3 biologically independent experiments). (C) Representative pictures showing eGFP expression in control and EPNs KD βIII-Tubulin::eGFP mESC cultures at 6 and 7 days of neuralization.


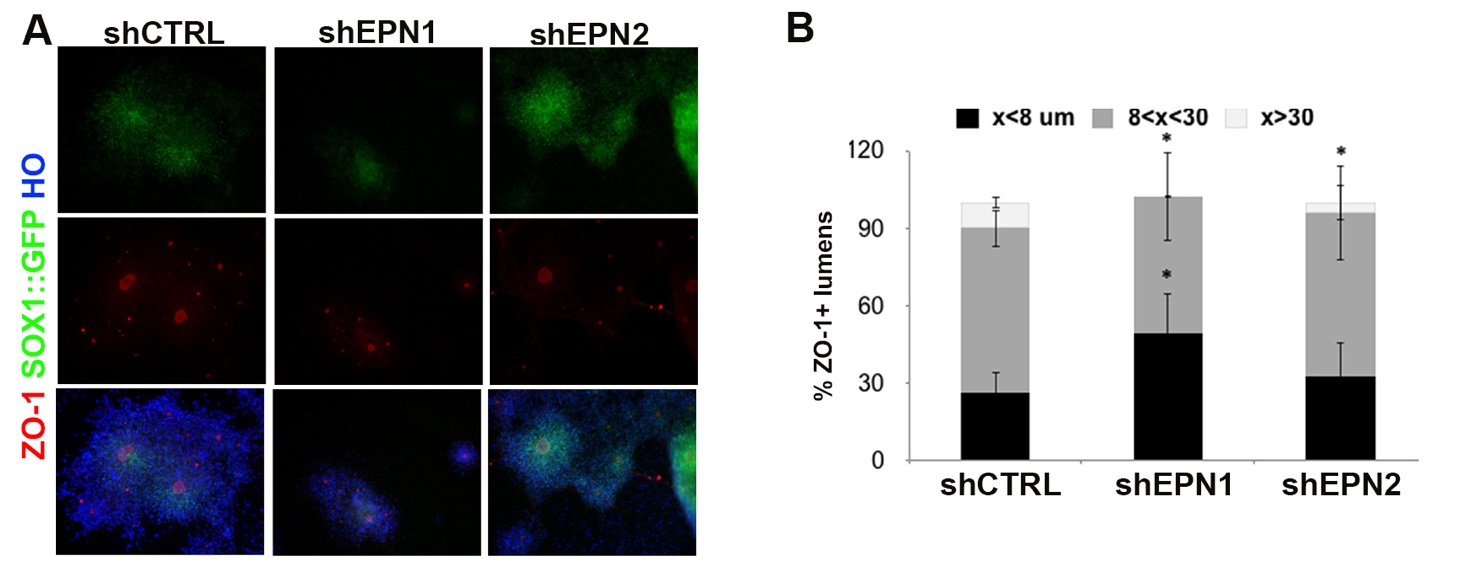


**Supplementary figure 7.** **EPN1 and EPN2 KD affect neural rosettes lumen size in neuralized mESC cultures.**

(A and B) 7 days neuralized shEPN1 46C mESC cultures are characterized by the complete absence of ZO-1 positive large-lumen rosettes (diameter greater than 30 μM), and a concomitant two-fold increase of small-lumen structures (diameter lower than 8 μM). Fewer differences are assessed for EPN2 KD cultures, as reported in the histogram. Nuclei are counterstained with Hoechst 33258. All data are expressed as the means ± STDV (n = 5 biologically independent experiments). Statistical significance (unpaired t-test): *p < 0.05, **p<0.001.

**SUPPLEMENTARY MATERIAL: Original Western Blot Scans Manuscript** **4084351.v2**

**Original Blots Figure 1A.**








**EPN2**

**EPN1**

**β-Actin**

**Original Blots Figure 2A.**






**β-Actin**

**Clathrin**

**Original Blots Figure 2C.**






**NICD**

**β−Actin**

**Original Blots Figure 3E.**




**E-cad**

**α-Tubulin**

**Original Blots Supplementary Figure 1C.**



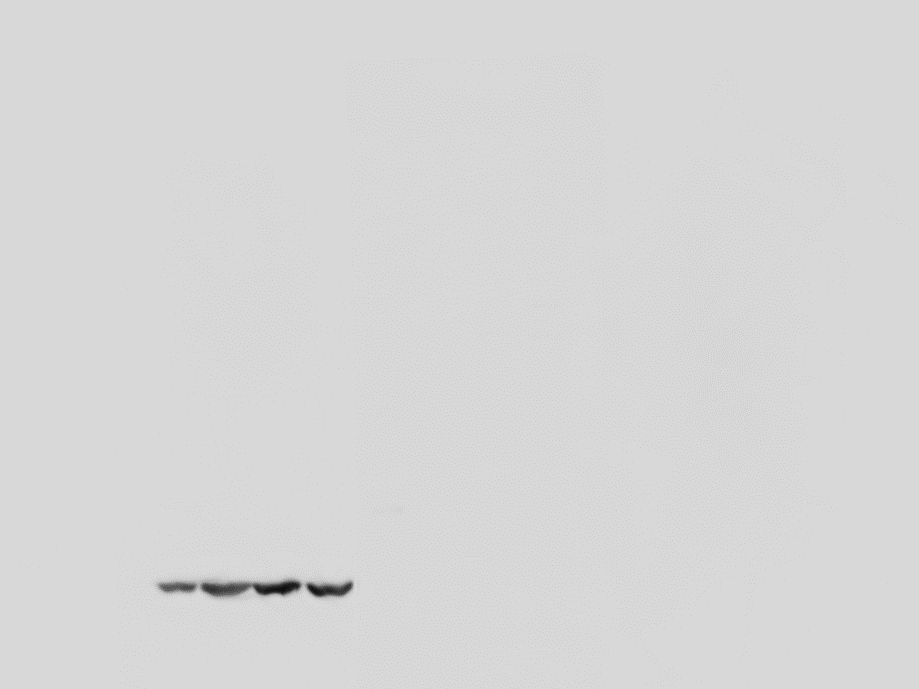


**β-Actin**

**Notch1**

**Original Blots Supplementary Figure 2A.**








**β-Actin**

**EPN1**

**EPN2**

**Original Blots Supplementary Figure 3A.**






**EPN2**

**α-Tubulin**


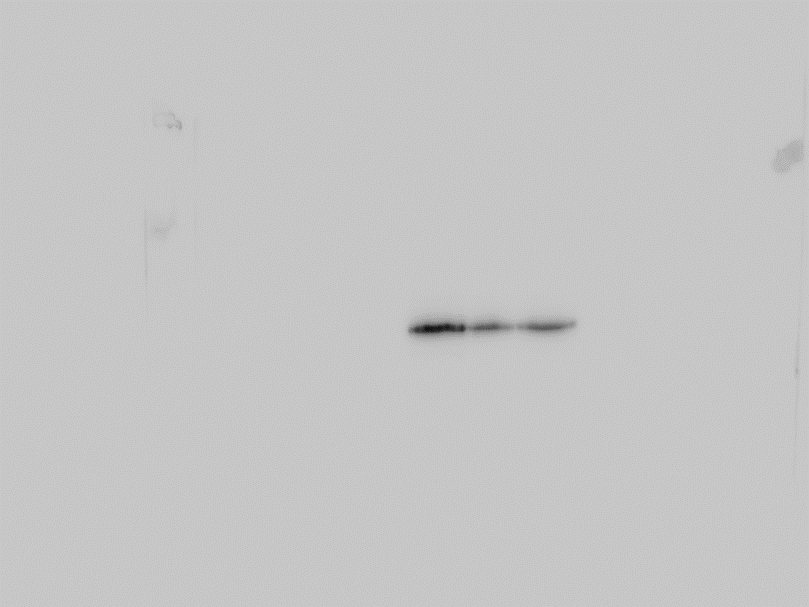


**EPN1**

**Original Blots Supplementary Figure 4C.**



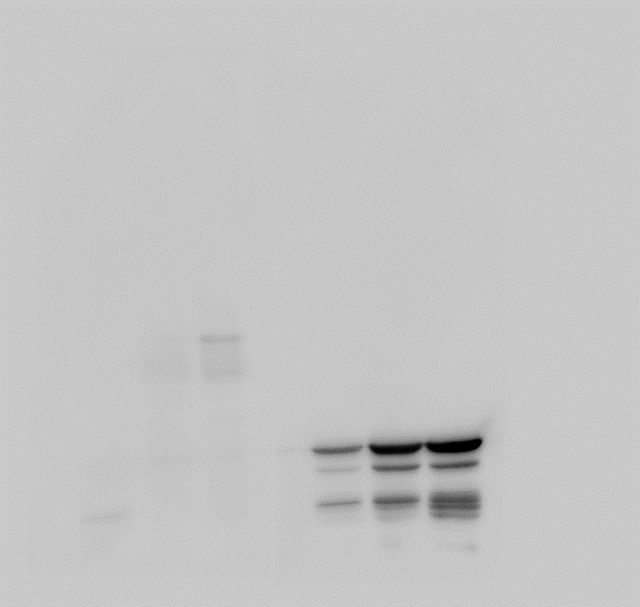

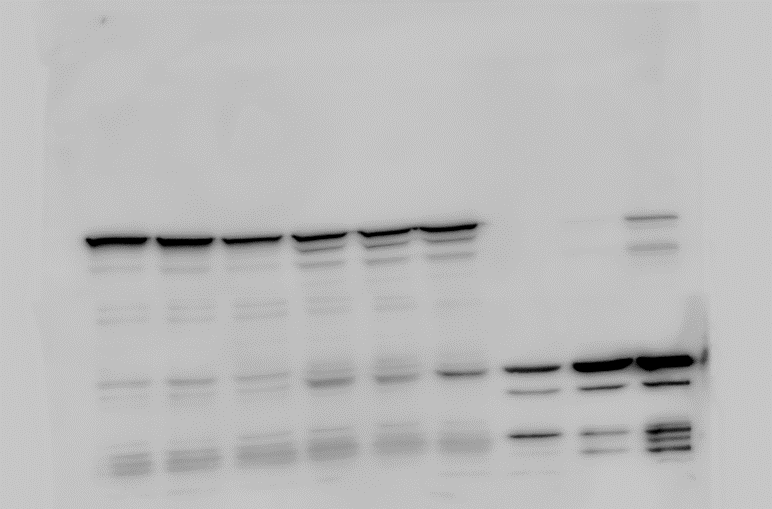




**α-SMA**

**SOX-17**

**CK-8**

**βIII-Tubulin**





**β-Actin**

**Original Blots Supplementary Figure 6B.**






**GFAP**

**βIII-Tubulin**





**α-Tubulin**
